# Loss of CARD9-mediated innate activation attenuates severe influenza pneumonia without compromising host viral immunity

**DOI:** 10.1038/srep17577

**Published:** 2015-12-02

**Authors:** Takayuki Uematsu, Ei’ichi Iizasa, Noritada Kobayashi, Hiroki Yoshida, Hiromitsu Hara

**Affiliations:** 1 Division of Molecular and Cellular Immunoscience, Department of Biomolecular Sciences, Faculty of Medicine, Saga University, 5-1-1 Nabeshima, Saga 849-8501, Japan; 2 Biomedical Laboratory, Department of Biomedical Research, Kitasato University Medical Center, 6-100 Arai, Kitamoto, Saitama 364-8501, Japan; 3 Department of Immunology, Graduate School of Medical and Dental Sciences, Kagoshima University, 8-35-1 Sakuragaoka, Kagoshima 890-8544, Japan

## Abstract

Influenza virus (IFV) infection is a common cause of severe viral pneumonia associated with acute respiratory distress syndrome (ARDS), which is difficult to control with general immunosuppressive therapy including corticosteroids due to the unfavorable effect on viral replication. Studies have suggested that the excessive activation of the innate immunity by IFV is responsible for severe pathologies. In this study, we focused on CARD9, a signaling adaptor known to regulate innate immune activation through multiple innate sensor proteins, and investigated its role in anti-IFV defense and lung pathogenesis in a mouse model recapitulating severe influenza pneumonia with ARDS. We found that influenza pneumonia was dramatically attenuated in *Card9*-deficient mice, which showed improved mortality with reduced inflammatory cytokines and chemokines in the infected lungs. However, viral clearance, type-I interferon production, and the development of anti-viral B and T cell immunity were not compromised by CARD9 deficiency. Syk or CARD9-deficient DCs but not macrophages showed impaired cytokine but not type-I interferon production in response to IFV *in vitro*, indicating a possible role for the Syk-CARD9 pathway in DCs in excessive inflammation of IFV-infected lungs. Therefore, inhibition of this pathway is an ideal therapeutic target for severe influenza pneumonia without affecting viral clearance.

Throughout history, worldwide pandemics of influenza have arisen and caused numerous deaths^1^,^2^,^3^,^4^,^5^. Primary influenza viral pneumonia (PIVP), the most severe pulmonary manifestation of influenza, is caused by the proliferation and activation of influenza virus (IFV) in the lungs. The incidence of PIVP was relatively high in past outbreaks, such as the 2009 pandemic of the H1N1 virus and avian influenza A/H5N1 virus infection. Many cases of PIVP develop into acute respiratory distress syndrome (ARDS) that is very difficult to treat and is associated with a high mortality rate^6^,^7^. While many cases of concomitant bacterial infection are successfully treated with antibiotics, there is no effective treatment for PIVP. Although vaccinations and anti-viral agents are effective against influenza, they cannot necessarily achieve remission in severe cases. Corticosteroids are often administered to patients in such cases, despite the lack of evidence for a beneficial effect, as well as the risk of unfavorable effects on viral replication^1^,^8^,^9^. Therefore, a strategy that does not rely on existing therapeutic concepts is required. Recent studies have demonstrated that excessive activation of the host’s innate immune system in response to the virus is a major factor in increasing the severity of influenza^10^,^11^,^12^. At the onset of PIVP, the invading virus triggers the host’s innate immune system including macrophages and dendritic cells (DCs) to secrete inflammatory cytokines/chemokines, such as IL-6, TNF, CXCL1 and CXCL10^13^,^14^, which play crucial roles in the pathogenesis of ARDS^10^,^15^,^16^,^17^. Therefore, inhibition of cytokine/chemokine production via targeting the host’s innate immune system may lead to the development of effective treatment options for ARDS.

Caspase recruitment domain family member 9 (CARD9) is an adaptor protein that delivers NF-κB activating signals through multiple innate sensor proteins, such as a wide array of C-type lectin (CLR) and immunoglobulin (Ig)-superfamily receptors that are coupled to immunoreceptor tyrosine-based activation motifs (ITAMs)^18^,^19^,^20^, as well as cytoplasmic RNA sensors, such as RIG-I and Mda5, and the DNA sensor Rad50^21^,^22^. Several lines of evidence have implied a role for CARD9-mediated innate activation pathways in influenza pathogenesis and immunity. The CLR dendritic cell-specific ICAM-3 grabbing non-integrin (DC-SIGN), which has a cytoplasmic ITAM-like sequence, was shown to bind influenza virus A^23^,^24^. Additionally, CARD9 was found to be required for the production of inflammatory cytokines after infection with RNA viruses, such as vesicular stomatitis virus and encephalomyocarditis virus, by regulating NF-κB signaling through RIG-I and Mda5^21^. However, the roles of CARD9 in influenza pneumonia, as well as in protection against IFV are yet to be elucidated.

In this study, we demonstrate a role for CARD9-mediated innate activation in influenza pathogenesis and immunity. Our results showed that the CARD9 pathway was involved in fatal influenza pneumonia mediated by inflammatory cytokine/chemokine production, whereas it was dispensable for type-I interferon production as well as the development of anti-viral acquired immunity. Therefore, inhibition of this pathway may represent an ideal target for the treatment of severe influenza pneumonia without affecting viral clearance.

## Results

### CARD9 deficiency attenuates lethal influenza pneumonia

**Figure 1:**
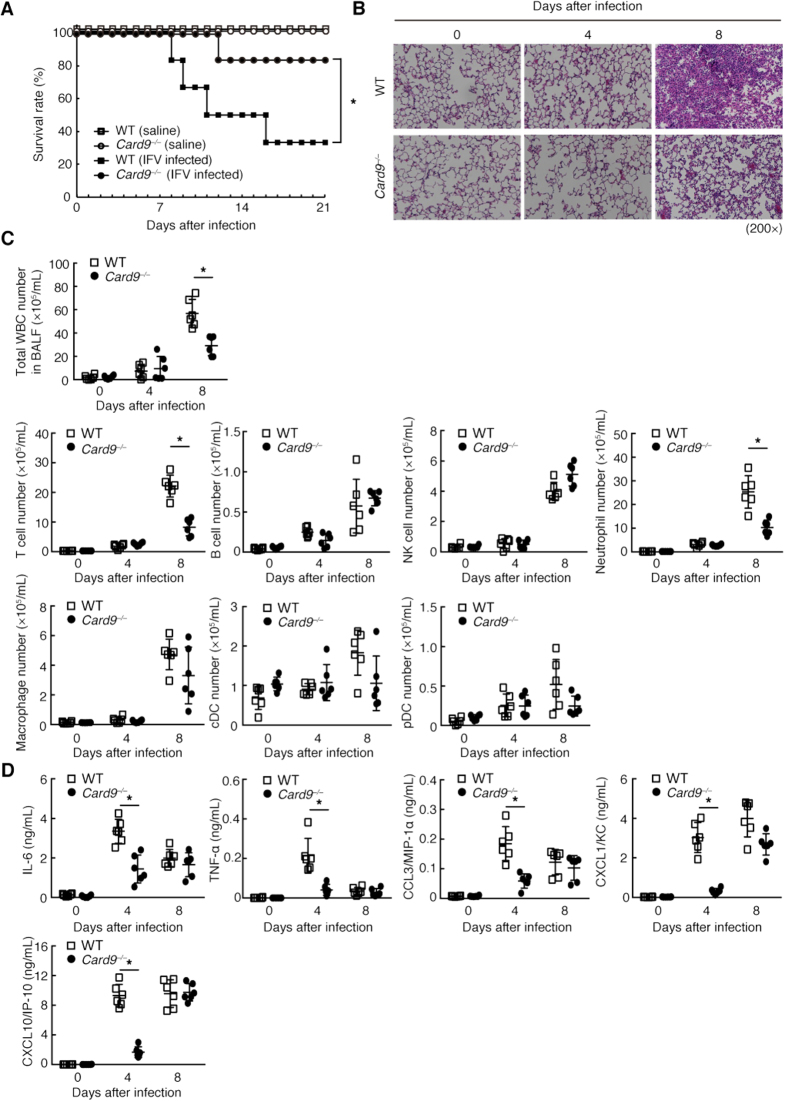
Loss of CARD9 attenuates severe influenza pneumonia. (**A**) Survival rate of mice after IFV infection. WT and *Card9*^*–/–*^ mice (n = 12 per group) were intratracheally infected with 10^4^ PFU of PR8 or injected with saline (sham-operated), followed by the assessment of survival rates. *P < *0.05* by log-rank test. (**B**) Hematoxylin and eosin staining of lungs on days 0, 4, and 8 after PR8 infection. Original magnifications, ×200. (**C,D**) Analysis of the BALF collected from WT and *Card9*^*–/–*^ mice (n = 6 per group) on day 0, 4, and 8 after PR8 infection for infiltrated cells (**C**) and inflammatory cytokine/chemokine levels (**D**). CD45^+^ white blood cells (WBC) in BALF were analyzed for T cells (CD3ε^+^ CD19^–^NK1.1^–^), B cells (CD19^+^ CD3ε^–^NK1.1^–^), NK cells (NK1.1^+^ CD3ε^–^CD19^–^), neutrophils (Ly-6 G^+^ F4/80^–^), macrophages (Ly-6 G^–^F4/80^+^), cDC (CD11c^hi^PDCA-1^–^Siglec-H^–^) and pDC (CD11c^low^B220^hi^PDCA-1^+^ Siglec-H^+^) by flow cytometry and the numbers of total and each cell populations per lung were calculated. Cytokine/chemokine levels in BALF were measured by a multiplex assay. Data are presented as mean ± SD, and are representative of three independent experiments. *P < *0.05* by Student’s t-test.

Because infection of the mouse-adapted IFV A/PR/8/34 strain (PR8) shows similar lung pathology to human ARDS^10^,^11^,^12^, we intratracheally infected wild-type (WT: C57BL/6) and *Card9*^*–/–*^mice with a lethal dose (10^4^ PFU/mouse) of PR8 to determine whether CARD9-mediated innate immune responses contributed to severe influenza pneumonia. We observed that PR8-infected WT mice appeared visibly ill with ruffled fur and reduced oral intake during the 6 to 10 days after infection, whereas *Card9*^*–/–*^mice appeared more active than WT mice. Consistent with their activity, the final survival rate up to day 21 after infection was dramatically improved in *Card9*^*–/–*^mice (~80%) as compared with WT mice (~40%; Fig. 1A). Histopathological analysis of IFV-infected lungs on days 4 and 8 revealed that lung inflammation, which was the most obvious on day 8 in WT mice, was much less severe in *Card9*^*–/–*^mice (Fig. 1B).

Given that inflammatory cytokines/chemokines were linked to lung damage in severe influenza pneumonia^10^,^11^,^12^,^15^,^16^,^17^,^25^, we analyzed infiltrated cells and cytokine/chemokine levels in bronchoalveolar lavage fluid (BALF) collected from the IFV-infected lungs of WT and *Card9*^*–/–*^ mice. Consistent with the pathological data, the number of CD45^+^ BALF cells were significantly fewer in the lungs of *Card9*^*−/−*^ mice than in those of WT mice on day 8, with a marked decrease in the numbers of T cells and neutrophils (Fig. 1C) that were the two main infiltrated subpopulations at this time point (Supplementary Fig. S1). However, the numbers of B cells, NK cells, macrophages, cDCs, and pDCs were not significantly affected by the CARD9 deficiency. The BALF levels of inflammatory cytokines and chemokines, i.e., IL-6, TNF-α, CCL3/MIP-1α, CXCL1/KC, and CXCL10/IP-10, which have been reported to contribute to lung pathology^10^,^13^,^14^, peaked on day 4 and were considerably lower in the *Card9*^*−/−*^ mice than in the WT mice (Fig. 1D). However, no reduction in cytokine/chemokine levels was evident on day 8, indicating that a CARD9-mediated innate response controls cytokine/chemokine production at an early time point after IFV infection and influences subsequent inflammatory cell recruitment and lung pathology at a later time point. Collectively, the loss of CARD9 attenuated severe influenza pneumonia and improved host mortality.

### CARD9 deficiency does not compromise anti-viral protective immunity

**Figure 2:**
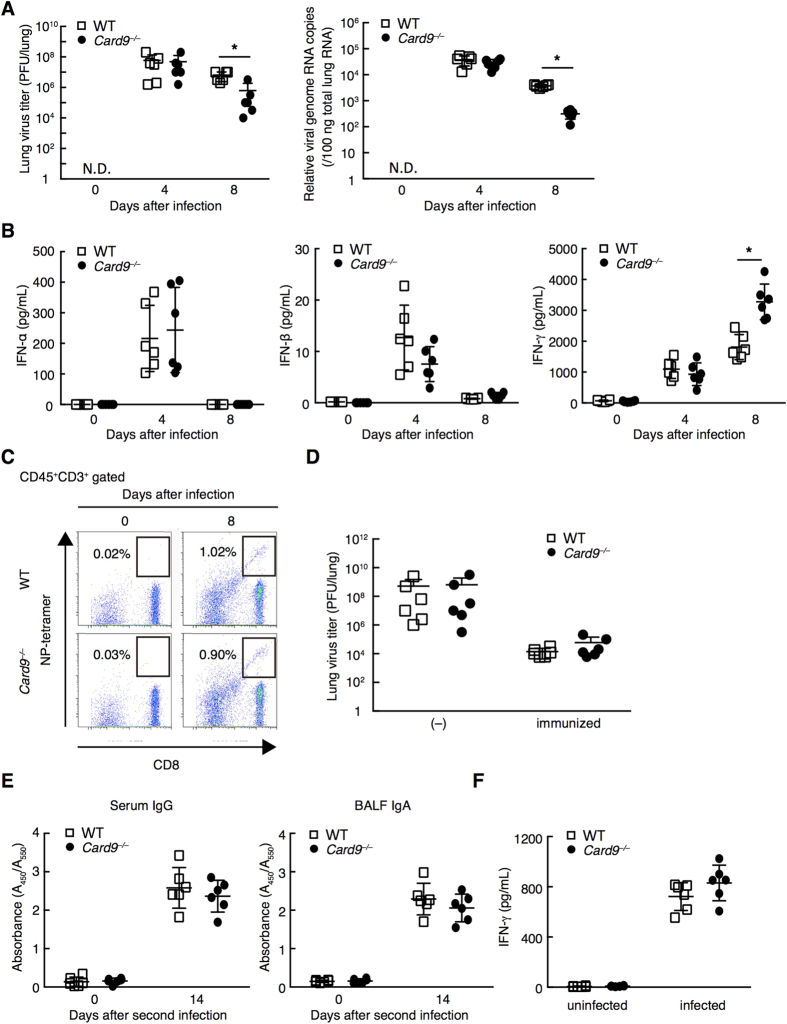
CARD9 deficiency does not affect induction of type-I interferons and host anti-viral response. (**A**) Viral titers and copy numbers in the infected lungs. The day 0, 4, and 8 lungs of WT and *Card9*^*–/–*^ mice (n = 6 per group) infected with PR8 were homogenized and the virus titers were quantified by a plaque assay in MDCK cells. Total RNA was extracted from the infected lungs and viral genome RNA copies were quantified by qPCR. (**B**) IFN-α, IFN-β and IFN-γ levels in the BALF from WT and *Card9*^*–/–*^ mice (n = 6 per group) after PR8 infection. Data are presented as mean ± SD, and are representative of three independent experiments. *P *<* *0.05* by Student’s t-test. (**C**) NP-specific CD8^+^ T cells in the mediastinal lymph nodes (MLN) after PR8 infection. The MLN cells of WT and *Card9*^*–/–*^ mice (n = 6 per group) were stained with anti-CD8α, anti-CD45 and anti-CD3ε mAbs, and H-2D^b^-NP_366–374_ tetramer and analyzed by flow cytometry. Representative dot plots are shown after gating on CD45^+^ CD3ε^+^ cells. The numbers near the outlines indicate the percentages of tetramer-specific cells. (**D**) Virus titers in the lungs of pre-immunized WT and *Card9*^*–/–*^ mice (n = 6 per group) two days post-challenge with a high lethal dose (10^6^ PFU: 100 LD_50_) of PR8. Mice were left unimmunized or immunized with a sublethal dose (10^3^ PFU: 1/10 LD_50_) of PR8 infection 28 days before challenge. (**E**) IFV-specific antibody production. WT and *Card9*^*–/–*^ mice (n = 6 per group) were infected twice with 10^3^ PFU of PR8 virus on day 0 and 21. IFV-specific IgG in serum and IgA in the BALF were analyzed on day 0 and 14 after the second infection. (**F**) IFV-specific adaptive T cell response. Splenocytes from WT and *Card9*^*–/–*^ mice (n = 6 per group) 14 days after infection as in (**E**) were stimulated *in vitro* with IFV nucleoprotein peptide (NP_366-374_) for 3 days. The culture supernatants were analyzed for IFN-γ production. Data are presented as mean ± SD of triplicates. Data are representative of three independent experiments. *P *<* *0.05* by Student’s t-test.

Activation of innate immunity in response to IFV regulates anti-viral protective immunity^26^. To evaluate the impact of CARD9 deficiency on anti-IFV protection, we first analyzed the viral burden in the IFV-infected lungs of WT and *Card9*^*–/–*^mice. Notably, we found that CARD9 deficiency did not increase virus titers and the amount of viral RNA in the lungs, but instead significantly accelerated viral clearance in the later stage (day 8) of infection (Fig. 2A). Type-I interferons (IFN-α/β) are the primary factors that induce host resistance to IFV^26^,^27^. In contrast to cytokine/chemokine production, CARD9 deficiency did not significantly affect IFN-α/β production in IFV-infected lungs (Fig. 2B). The type II interferon IFN-γ is not essential for viral clearance^28^, but its presence during IFV infection ameliorates the severity of inflammation and lung injury^29^. IFN-γ production was significantly more in the lungs of *Card9*^*−/−*^ mice than in those of WT mice on day 8 but not on day 4 (Fig. 2B); thus, this increase might contribute to the improvement of lung pathology in *Card9*^*−/−*^ mice. Moreover, the expression of IP-10 encoded by an IFN-responsive gene was significantly impaired in *Card9*^*−/−*^ mice on day 4 (Fig. 1D), albeit no significant reduction was seen in either Type I or Type II interferons, suggesting that synergistic signals of interferons and various cytokines/chemokines are required for the higher expression of IP-10 at an early phase of infection. Next, we examined the induction of virus-specific CD8^+^ T cells using the H-2D^b^ tetramer coupled with a viral nuclear protein (NP)-derived peptide (NP_366–374_)^30^,^31^. The percentages of NP-specific CD8^+^ T cells in the draining lymph nodes of the lungs on day 8 were comparable between WT and *Card9*^*−/−*^ mice (Fig. 2C). To evaluate the impact of CARD9 deficiency on acquired immunity to IFV, WT and *Card9*^*−/−*^ mice were immunized with a sub-lethal dose (1/10 of LD_50_) of the PR8 strain and rechallenged with a high lethal dose (100 LD_50_) of PR8 after 4 weeks. We found that CARD9 deficiency did not alter viral clearance after the challenge (Fig. 2D). Next, we assessed the induction of humoral and cellular acquired immunity to IFV in *Card9*^*–/–*^mice. We re-infected WT and *Card9*^*–/–*^mice on day 21 after their first infection (representing the second infection) and analyzed the production of virus-specific antibodies as well as the development of virus-specific CD8^+^ T cells on day 14 after the second infection. We found that the CARD9 deficiency affected neither the production of virus-specific IgG in the serum nor virus-specific IgA in the lung mucosa (Fig. 2E). Additionally, CARD9 deficiency did not affect the IFN-γ response by splenic CD8^+^ T cells specific to a NP antigen epitope NP_366–374_ (Fig. 2F). Collectively, the CARD9 pathway is dispensable for the induction of protective immunity against both the primary and secondary IFV infections, and its deficiency even improves primary viral clearance upon lethal-dose infections probably because of improvement in lung damage and elevated IFN-γ production.

### CARD9 deficiency impairs inflammatory cytokine, but not type-I interferon, production by DCs

**Figure 3:**
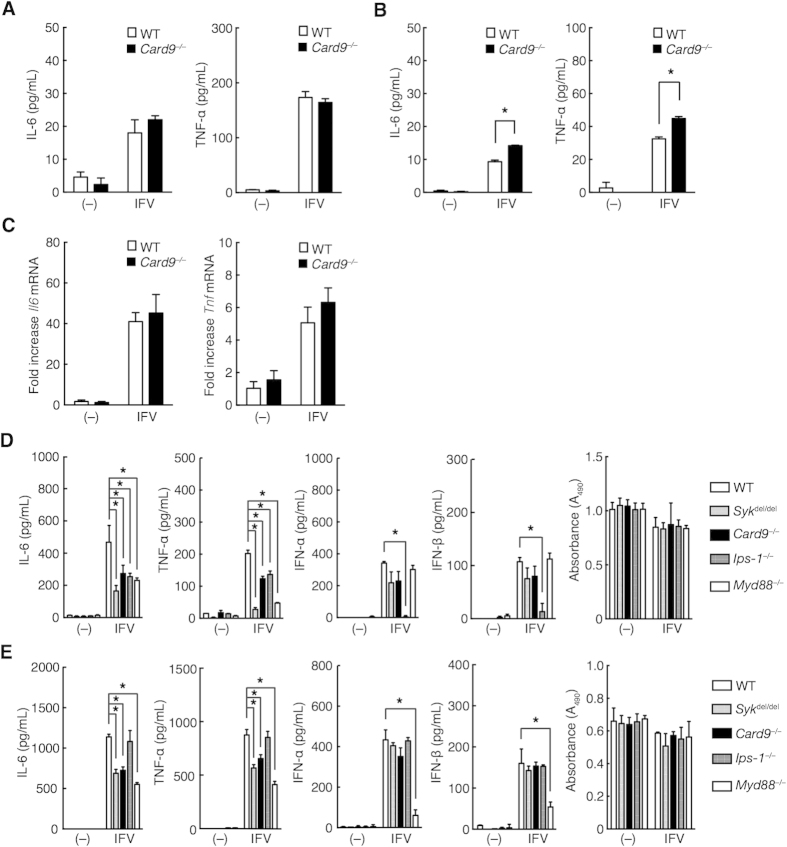
CARD9 pathway controls inflammatory cytokine but not type-I interferon production in response to IFV by DCs but not MFs. (**A,B**) IL-6 and TNF-α productions by WT or *Card9*^*–/–*^TG-MFs (**A**) and BMMFs (**B**) after stimulatio with PR8 for 24 h. (**C**) *Il6* and *Tnf* mRNA expressions by WT or *Card9*^*–/–*^AMFs after stimulation with PR8 for 6 h. (**D,E**) IL-6, TNF-α, IFN-α and IFN-β productions by WT, *Syk*^del/del^*, Card9*^*–/–*^, *Ips-1*^*−/−*^ or *Myd88*^*−/−*^bone marrow-derived cDCs (**D**) and FLT3L-DCs (**E**) after stimulation with PR8 for 24 h. Cytokine and interferon levels in the culture supernatants were measured by ELISA. The cellularity/viability of cDC and FLT3L-DCs were determined by MTS assay after the stimulation and indicated in the rightmost histograms in (**D,E**), respectively. Data are presented as mean ± SD of triplicates, and are representative of three independent experiments. *P < *0.05* by Student’s t-test.

**Figure 4:**
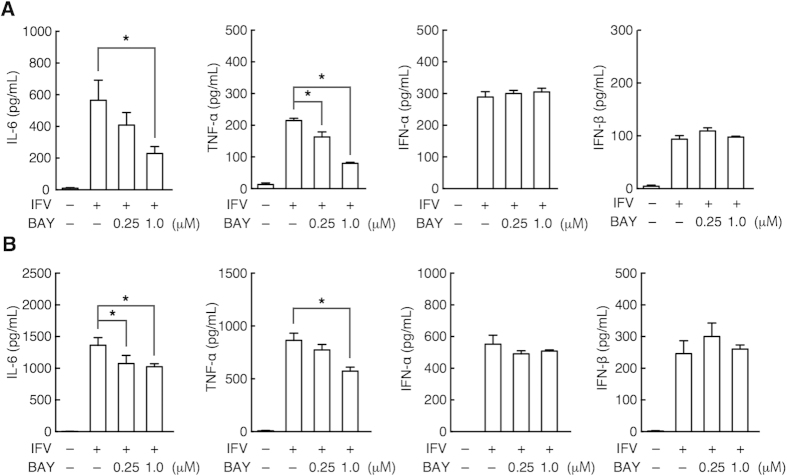
Specific inhibition of inflammatory cytokine but not type-I interferon production in response to IFV by a Syk inhibitor. WT cDCs (**A**) and FLT3L-DCs (**B**) were pretreated for 1 h with control vehicle (–) or indicated amounts of BAY61-3606 (BAY), and then stimulated with PR8 for 24 h. IL-6, TNF-α, IFN-α and IFN-β levels in the cell culture supernatants were measured by ELISA. Data are presented as mean ± SD of triplicates, and are representative of three independent experiments. *P < *0.05* by Student’s t-test.

Because macrophages and DCs are known to be an early source of inflammatory cytokines and type-I interferons in pulmonary IFV infection^13^,^14^, we examined whether CARD9 was required for their production by myeloid cells in response to IFV. Thioglycolate-induced peritoneal macrophages (TG-MFs), bone marrow-derived macrophages (BMMFs), alveolar macrophages (AMFs), conventional DCs (cDCs), or Flt3 ligand-induced plasmacytoid DCs (FLT3L-DCs) prepared from WT or *Card9*^*–/–*^mice were brought into contact with PR8 *in vitro* and the production of IL-6, TNF-α, and IFN-α/β was measured. IL-6 and TNF-α produced by TG-MFs (Fig. 3A) or BMMFs (Fig. 3B) and those mRNAs produced by AMFs (Fig. 3C) in response to PR8 were not affected by CARD9 deficiency. As previously reported^18^,^19^, CARD9 was required for a cytokine response to ox-zymosan through Dectin-1-Syk but dispensable for the response to LPS through TLR4-MyD88 in cDCs (Supplementary Fig. S2A). We found that *Card9*^*–/*–^ cDCs (Fig. 3D) or FLT3L-DCs (Fig. 3E) produced significantly lower levels of IL-6 and TNF-α than WT cells in response to PR8. In contrast, IFN-α/β production was not compromised in *Card9*^*–/*–^ cDCs or FLT3L-DCs. These findings suggest that CARD9 deficiency primarily affected DCs but not MFs regarding the production of inflammatory cytokines but not IFN-α/β in response to IFV. It has been reported that the CARD9-IPS-1 pathway mediates the cytokine response to RNA viruses through RLHs^21^, while CARD9 has been found to transmit signals from myeloid ITAM-coupled receptors via Syk^18^,^19^,^20^. Thus, we evaluated the contributions of these pathways. IPS-1-deficient (*Ips-1*^–/–^) cDCs and Myd88-deficient (*Myd88*^–/–^) FLT3L-DCs produced significantly lower levels IFN-α/β than WT cells in response to PR8 (Fig. 3D,E), consistent with previous findings showing that the IFN-α/β response to RNA viruses is mediated mainly through RIG-I/IPS-1 in cDCs, and through TLR7/MyD88 in pDCs^32^,^33^. In addition, IL-6 and TNF-α production was impaired in *Ips-1*^–/–^ cDCs and *Myd88*^–/–^ FLT3L-DCs. Lower levels of cytokine production were also observed in *Myd88*^–/–^ cDCs, likely due to impaired activation through several TLRs (i.e. TLR3, 7, 8 and 9) that sense virus nucleic acids^34^,^35^. We found that inducible *Syk*-deficient (*Syk*^del/del^) cDCs and FLT3L-DCs produced significantly lower levels of inflammatory cytokines than WT cells similar to the responses of *Card9*^*–/*–^ cDCs and FLT3L-DCs, whereas the Syk deficiency did not affect cytokine production in response to LPS (Supplementary Fig. S2A). Consistent with these findings, treatment of WT cDCs (Fig. 4A) and FLT3L-DCs (Fig. 4B) with the Syk inhibitor BAY61-3606 reduced the production of inflammatory cytokines but not type-I interferons in response to PR8 in a dose-dependent manner. The treatment with BAY61-3606 reduced the cytokine response to ox-zymosan but not to LPS, indicating that the effect of the inhibitor on cytokine expression depends on the stimulus but cannot be attributed to its general effect on cytokine gene expression (Supplementary Fig. S2B). Overall, these results suggest that the attenuation of influenza pneumonia by *Card9* deficiency was likely attributable to the reduced cytokine/chemokine production by pulmonary DCs through the Syk-CARD9 pathway in response to IFV.

## Discussion

We have shown that CARD9-mediated activation of the innate immune system exacerbates influenza pneumonia in mice. CARD9 deficiency resulted in the reduction of inflammatory cytokine/chemokines and the infiltration of inflammatory cells in IFV-infected lungs, resulting in improved mouse mortality rates. Given the pathological similarity between the mouse intratracheal infection model and influenza-associated ARDS in human^10^,^11^,^12^, we postulate that the CARD9 pathway contributes to the exacerbation of human influenza pneumonia. Although the Syk-CARD9-mediated innate immune response is crucial for anti-fungal acquired immunity^36^,^37^,^38^,^39^, our data showed that CARD9 was dispensable for anti-IFV immunity, as demonstrated by the findings that CARD9 deficiency did not alter viral burden, the elevation of IFN-α/β, or the induction of anti-viral adaptive T and B cell responses in the IFV-infected mice. Thus, other innate mechanisms independent of the CARD9 pathway may play a more dominant role in protective immunity against IFV.

Innate immune sensors for IFV and its downstream signaling pathway have been well illustrated^26^,^40^,^41^. In our study, CARD9 deficiency resulted in a selective reduction in inflammatory cytokines, but not IFN-α/β production, by both cDCs and FLT3L-DCs in response to IFV. This pattern of impairment was observed in *Ips-1*^–/–^ cDCs, and was consistent with results from previous reports indicating that CARD9 selectively regulates the cytokine response through RIG-I-IPS-I in cDCs^21^. Additionally, we found that *Syk*^del/del^ and Syk inhibitor-treated cDCs showed the same pattern of impairment as *Card9*^–/–^ or *Ips-1*^–/–^ cDCs. On the other hand, in FLT3L-DCs known to release large amounts of inflammatory cytokines and type-I interferons upon recognition of IFV^32^,^33^, IPS-1 deficiency affected neither cytokine or type-I interferon responses. However, Syk deficiency in FLT3L-DCs, similar to that in cDCs, resulted in a selective reduction in inflammatory cytokines but not IFN-α/β. Thus, it is likely that the Syk-CARD9 pathway controls detrimental cytokine production by pulmonary DCs upon acute influenza infection. Alternatively, because CARD9 deficiency impairs cytokine production by DCs following stimulation with several TLR ligands^18^, it may be important to examine whether the Syk-CARD9 pathway is involved in the TLR7-meditated response to IFV.

The involvement of the Syk-CARD9 pathway implies the presence of ITAM-coupled receptors that sense IFV and stimulate cytokine production by DCs. It has been reported that an interaction between DC-SIGN, which possesses an ITAM-like “hemITAM” motif, and the carbohydrate of IFV hemagglutinin induces maturation of DCs and promotes endocytosis of the virus^42^, indicating the signal-activating capacity of DC-SIGN upon virus binding. However, it is unclear whether the DC-SIGN hemITAM is capable of transmitting sufficient signals to produce inflammatory cytokines. With regard to other viruses, recognition of the dengue virus by the DAP12-associated CLR CLEC5A results in increased vascular permeability due to the overproduction of TNF-α, resulting in fatal outcomes^43^. However, its ability to recognize the IFV remains unknown. Thus, it is worth examining whether these CLRs are involved in the cytokine response to IFV. It is also conceivable that damage-associated molecular patterns (DAMPs), generated due to lung damage by IFV infection, may be ligands for some ITAM-coupled receptors. Indeed, the FcRγ-associated CLR Mincle was shown to recognize SAP130, a component of the ribonucloprotein released from dead cells^44^.

In conclusion, CARD9-mediated innate immune activation in pulmonary DCs acts to exacerbate severe influenza pneumonia, but is dispensable for host protection against IFV. Thus, inhibiting the CARD9 pathway may represent a promising therapeutic target for the control of PIVP without affecting viral clearance.

## Methods

### Mice

*Card9*^*–/–*^, *Sy*k^flox/flox^, *Ips-1*^*–/–*^and *Myd88*^*–/–*^mice have been previously described^18^,^45^,^46^,^47^. These mice were backcrossed at least 8 times onto C57BL/6 mice. C57BL/6 mice were purchased from CLEA Japan, Inc. (Tokyo, Japan). The animals were housed in specific pathogen-free conditions. All experiments were approved by the Institutional Animal Care and Use Committee for Kitasato University Medical Center and animals were treated in accordance with the Regulations for Animal Experiments in Kitasato University. All surgeries were performed under ketamine hydrochloride/xylazine anesthesia, and all efforts were made to minimize suffering.

### Antibodies and reagents

Fluorescein isothiocyanate (FITC)-conjugated anti-F4/80 (clone BM8), FITC-conjugated anti-CD45R/B220 (clone RA3-6B2), FITC-conjugated CD19 (clone 6D5), Phycoerythrin (PE)-conjugated anti-Ly-6G (clone 1A8), PE-conjugated anti-SiglecH (clone 551), biotin-conjugated anti-NK1.1 (clone PK136), biotin-conjugated anti-BST2/PDCA-1 (clone 927), peridinin chlorophyll protein/Cy5.5 (PerCP/Cy5.5)-conjugated anti-CD45 (clone 30-F11), phycoerythrin-Cy7 (PC7)-conjugated anti-CD3ε (clone 145-2C11) and PC7-conjugated anti-CD11c (clone N418) monoclonal antibodies were purchased from Biolegend (San Diego, CA). FITC-conjugated anti-CD8 (clone KT15) monoclonal antibodies, PE-conjugated H-2D^b^ tetramer coupled with a viral NP-derived ASNENMETM peptide (NP_366-374_: ASNENMETM), H-2D^b^-restricted influenza NP_366-374_ peptide were purchased from MBL (Nagoya, Japan). Syk inhibitor IV (BAY 61–3606) was purchased from Merck Millipore (Billerica, MA). LPS from *Escherichia coli* 0111:B4 was purchased from Sigma-Aldrich (St. Louis, MO). NaClO-oxidized zymosan was prepared as described^48^.

### IFV infection in mice

C57BL/6 and *Card9*^*–/–*^ mice were anesthetized and infected with 10^4^ plaque forming units (PFU) (unless otherwise indicated) of a mouse-adapted IFV (A/PR/8/34 strain: H1N1 isotype, kindly provided by the Kitasato Institute, Tokyo, Japan) by intratracheal administration as described^10^,^11^,^12^.

### Histology

Whole lungs were collected at 0, 4, and 8 days after IFV infection. Paraffin embedding and hematoxylin and eosin staining of tissues were performed using standard methodologies.

### Bronchoalveolar lavage (BAL)

BAL was carried out as described previously^25^,^49^. In Brief, tracheas of mice were cannulated with 1.2- mm diameter polyethylene catheters. Lungs were instilled with 1 mL of pre-warmed PBS containing 5 mM EDTA, followed by the retrieval of lavage fluid aliquots. Cells in the BAL fluid (BALF) were counted after red blood cell lysis and subjected to flow cytometric analysis. The supernatants of the BALF were subjected to multi cytokine/chemokine expression analysis and cytokine ELISA.

### Cytokine/chemokine expression

Cytokine/chemokine in the BALF was measured with a Flow Cytomix Cytokine Bead Assay (Bender MedSystems, Vienna, Austria), with the exception of IFN-β, which was measured by a VeriKine^TM^ Mouse Interferon-β ELISA kit (PBL InterferonSource, Piscataway, NJ). Cell culture supernatants from splenocytes were assayed using a specific ELISA kit for IFN-γ (Biolegend). Cell culture supernatants from TG-MFs, BMMFs, cDCs, and FLT3L-DCs were assayed using specific ELISA kits for IL-6, TNF-α (Biolegend), IFN-α and IFN-β (PBL InterferonSource). All measurements were performed in triplicate. For quantitative PCR (qPCR) analysis for cytokine mRNAs, total RNA was extracted from cells with ReliaPrep RNA Cell Miniprep System (Promega, Madison, WI) and cDNA was synthesized with PrimeScript RT reagent Kit (Takara Bio, Shiga, Japan) according to the manufacturer’s instructions. qPCR was performed with the Applied Biosystems 7900HT Fast Real Time PCR System (Life Technologies). Primer sequences were as follows: mouse *Il6*, forword, 5′- CCA CTT CAC AAG TCG GAG GCT TA - 3′, and reverse, 5′- GCA AGT GCA TCA TCG TTG TTC ATA C - 3′; and *Tnf* forword, 5′- GTT CTA TGG CCC AGA CCC TCA C - 3′, and reverse, 5′- GGC ACC ACT AGT TGG TTG TCT TTG - 3′.

### Flow cytometry

BALF cells and mediastinal lymph node cells were suspended in mouse FcR Blocking Reagent (Miltenyi Biotec, Bergisch Gladbach, Germany) for 10 min before staining with FITC-, PE-, biotin-, PerCP/Cy5.5- or PC7-conjugated antibodies or PE-conjugated H-2D^b^ tetramer. After staining, biotinylated antibodies were visualized with streptavidin-energy-coupled dye (Beckman Coulter, Fullerton, CA). The antibodies used were anti-F4/80, anti-CD45R/B220, anti-CD8, anti-CD19, anti-Ly-6G, anti-Siglec H, anti-NK1.1, anti-BST2/PDCA-1, anti-CD45, anti-CD3ε and anti-CD11c. Stained cells were analyzed with a Cytomics FC500 flow cytometer (Beckman Coulter) and Flowjo software (Tree Star, Ashland, OR).

### Virus titer in the lungs

Mice were euthanized by intraperitoneal administration of sodium pentobarbital at 0, 4, or 8 days after IFV infection. Lung tissues were homogenized using a gentleMACS dissociator (Miltenyi Biotec), and the homogenates were serially diluted with cold PBS. For a plaque assay, Madin-Darby canine kidney (MDCK) cells (obtained from JCRB cell bank, Osaka, Japan) were plated at 1 × 10^6^ cells in a flat-bottomed 6-well plate (Corning, Corning, NY) a day before infection. Supernatants from lung homogenates serially diluted were used to infect the MDCK cells at 37 °C for 1 h. The cells were subsequently overlaid with DMEM (Wako Pure Chemical Industries, Osaka, Japan) mixed with 0.75% noble agar (Difco, Detroit, MI) in the presence of 2 μg/mL crystallized trypsin (Wako Pure Chemical Industries). The plaques were visualized by staining the cells with Methylene Blue (Nacalai tesque, Kyoto, Japan), and the cells were counted 4 days after infection.

### Viral copy numbers

Mice were euthanized by intraperitoneal administration of sodium pentobarbital at 0, 4, or 8 days after IFV infection. Lung tissues were homogenized using a gentleMACS dissociator (Miltenyi Biotec) and RNA was extracted with an Isogen II RNA extraction kit (Nippon Gene, Tokyo, Japan). Reverse transcription was conducted with the Uni-12 primer (5′- AGC AAA AGC AGG -3′)^50^ and qPCR was preformed with primers specific for NP (Forward: 5′- GAT TGG TGG AAT TGG ACG AT -3′; Reverse: 5′- AGA GCA CCA TTC TCT CTA TT -3′) using the Applied Biosystems 7900HT Fast Real Time PCR System (Life Technologies, Carlsbad, CA). The standard calibration curve for qPCR was obtained by stepwise dilution of the cloned NP gene fragment with a known copy number.

### IFV rechallenge

WT and *Card9*^*–/–*^ mice were left uninfected or infected intratracheally with sublethal dose (10^3^ PFU/mouse: 1/10 LD_50_) of PR8. Twenty-eight days after the first infection, mice were challenged with a high lethal dose (10^6^ PFU/mouse: 100 LD_50_) of PR8. Two days postchallenge, virus titers in the lungs were measured in a plaque assay in MDCK cells.

### Antigen-specific B cell responses

B cell–mediated humoral responses were measured as virion-specific immunoglobulin production by ELISA, as previously described^32^. Briefly, 96-well ELISA plates (Corning) were coated with ultrasonicated influenza virion (A/PR/8/34 strain) at 5 × 10^6^ PFU/mL in a carbonate buffer (pH 9.6), and incubated overnight at 4 °C. Plates were then washed with PBS containing 0.05% Tween 20 (Wako Pure Chemical Industries). Serum and BALF collected from mice at day 14 after the secondary infection were serially diluted with PBS/Tween 20 containing 5% skim milk, applied onto the virion-coated plates, and incubated for 2 h at room temperature. After washing, goat anti-mouse total IgG or IgA conjugated to horseradish peroxidase (Jackson Immunoresearch, Baltimore Pike, PA) was applied and incubated for 2 h at room temperature. After washing, the plates were stained with a TMB Substrate Set (Biolegend). The reaction was terminated with 1 M H_2_SO_4_ (Wako Pure Chemical Industries) and the absorbance was measured.

### Antigen-specific T cell responses

IFV-specific T cell responses were measured as viral NP– specific IFN-γ secretion by splenocytes, as described previously^32^,^51^. Briefly, 5 × 10^5^ splenocytes extracted from mice 14- days after the secondary infection were seeded on 96-well cell culture plates (Corning) and then stimulated with 10 μg/mL of NP_366–374_ peptides. After 3 days of culture, supernatants were collected and analyzed for IFN-γ production by ELISA (Biolegend).

### MFs/DCs preparation and IFV stimulation *in vitro*

TG-MFs were prepared as described^36^. AMFs were isolated from BALF of WT or *Card9*^*–/–*^ mice. BMMFs, cDCs, or FLT3L-DCs were prepared by culturing bone marrow cells for 5–8 days with RPMI1640 medium (Wako Pure Chemical Industries) supplemented with 10% fetal bovine serum (Life Technologies) and antibiotics (100 IU/mL penicillin and 100 μg/mL streptomycin; Sigma-Aldrich) containing M-CSF (25 ng/mL, Peprotech, Rocky Hill, NJ), GM-CSF (20 ng/mL, Peprotech) or human Flt3-Ligand (100 ng/mL, Peprotech), respectively. The FLT3L-DCs derived from the culture contain constantly 10–13% of PDCA-1^+^ SiglecH^+^ plasmacytoid DCs (pDCs) irrespective of deficiency in *Syk*, *Card9*, *Ips-1* or *MyD88*. For Syk deletion *in vitro*, cultured cells derived from *Syk*^flox/flox^ mice were incubated with 0.6 μM of the active metabolite of tamoxifen, 4-hydroxytamoxifen (Sigma-Aldrich) for 3 d. For stimulation of MFs/DCs of, 1 × 10^5^ cells were seeded on 24-well culture plates (Corning) and incubated overnight, followed by replacement of 200 μL of serum-free medium containing 10^6^ PFU (multiplicity of infection [M.O.I] = 10) of PR8. After 1 h of incubation, unabsorbed viruses were removed and the cells were incubated for a further 24 h in serum-containing medium. DCs were also stimulated with LPS (100 ng/mL) or NaClO-oxidized zymosan (10 μg/mL) for 24 h in serum-containing medium. The culture supernatants were assayed for IL-6, TNF-α, IFN-α, and IFN-β by ELISA. For stimulation of AMFs, 1 × 10^4^ cells were seeded on 96-well culture plate (Corning), stimulated as above with 10^5^ PFU (M.O.I = 10) of PR8 for 6 h, and total RNAs were extracted for qPCR analysis for cytokine expression. For treatment of cells with a chemical Syk inhibitor, WT DCs were incubated with 0.25 or 1 μM BAY 61–3606 for 1 h prior to virus stimulation.

### Cell viability

Cell viability was determined by the MTS assay using the CellTiter 96 AQueousOne Solution Cell Proliferation Assay kit (Promega). In brief, 20 μL of MTS solution was added to cells in 96-well plates after *in vitro* IFV stimulation and the plates were incubated for 2 h at 37 °C, then the absorbance at 490 nm was measured using a Benchmark microplate reader (Bio-Rad, Hercules, CA).

### Statistical analysis

Survival curves were generated by the Kaplan-Meier method, and statistical analyses were performed using the log-rank test. The statistical significance was assessed by Student’s t-tests. A *P* value < 0.05 was considered significant.

## Additional Information

**How to cite this article**: Uematsu, T. *et al.* Loss of CARD9-mediated innate activation attenuates severe influenza pneumonia without compromising host viral immunity. *Sci. Rep.* **5**, 17577; doi: 10.1038/srep17577 (2015).

## Supplementary Material

Supplementary Information
