# Supplementary material for: Loss of CARD9-mediated innate activation attenuates severe influenza pneumonia without compromising host viral immunity
Source: Sci Rep. 2015 Dec 2;5:17577. doi: 10.1038/srep17577 (PMC4667252; doi:10.1038/srep17577)
Supplement: Supplementary Information [file srep17577-s1.pdf]

Supplementary Information

Loss of CARD9-mediated innate activation attenuates severe influenza pneumonia without compromising host viral immunity

Takayuki Uematsu[1,2], Ei'ichi Iizasa[1,3], Noritada Kobayashi[2], Hiroki Yoshida[1], Hiromitsu Hara[1,3]*

[1]Division of Molecular and Cellular Immunoscience, Department of Biomolecular Sciences, Faculty of Medicine, Saga University, Saga 849-8501, Japan, [2]Biomedical Laboratory, Department of Biomedical Research, Kitasato University Medical Center, Saitama 364-8501, Japan, [3]Department of Immunology, Graduate School of Medical and Dental Sciences, Kagoshima University, Kagoshima 890-8544, Japan.

*Correspondence and requests for materials should be addressed to H.H. (harah@m2.kufm.kagoshima-u.ac.jp)

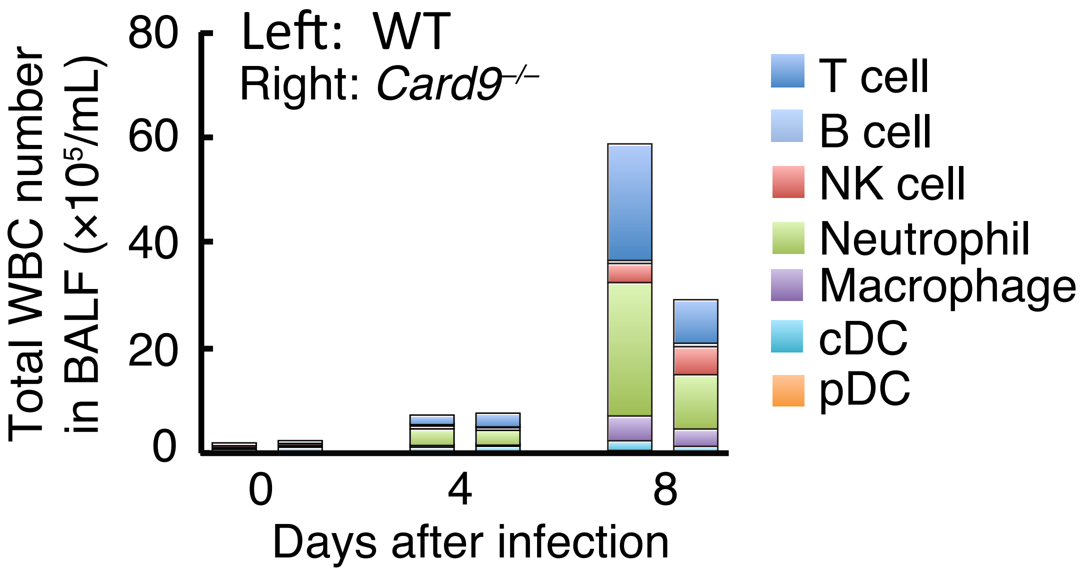

Supplementary Figure S1

Analysis of WBC subpopulations in BALF. T cells, B cells, NK cells, neutrophils, macrophages, cDCs and pDCs within $CD45^{+}$ white blood cells (WBC) in BALF collected from the lungs of WT and *Card9*$^{-/-}$ mice (n = 6 per group) at day 0, 4 and 8 after PR8 infection were analyzed by flow cytometry and their mean absolute numbers per lung were indicated as a stacked bar graph.

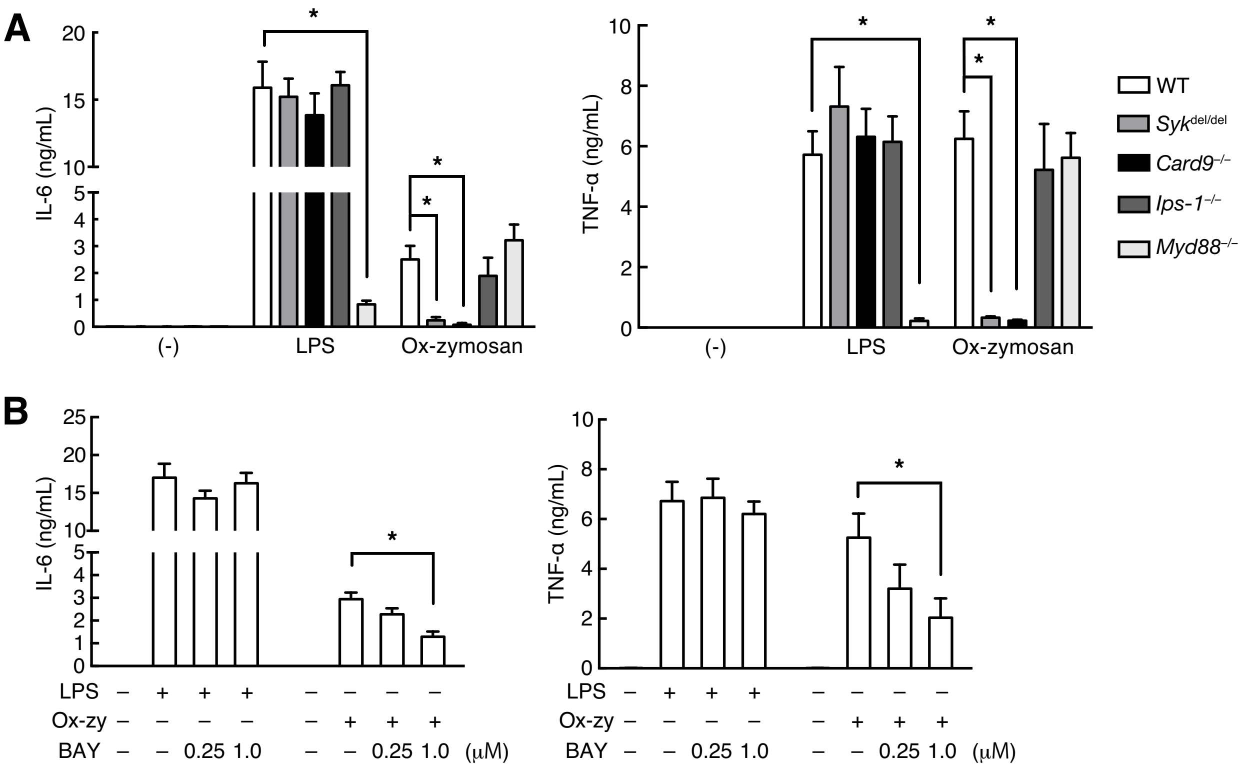

Supplementary Figure S2

(A) Inflammatory cytokine productions by WT, *Syk*$^{del/del}$, *Card9*$^{-/-}$, *Ips-1*$^{-/-}$ or *Myd88*$^{-/-}$cDCs in response to LPS or ox-zymosan. Bone marrow-derived cDCs prepared from WT, *Syk*$^{del/del}$, *Card9*$^{-/-}$, *Ips-1*$^{-/-}$ or *Myd88*$^{-/-}$ mice were stimulated *in vitro* with LPS (100 ng/mL) or NaClO-oxidized zymosan (ox-zymosan; 10μg/mL) for 24 h. IL-6 and TNF-α levels in the cell culture supernatants were measured by ELISA. Data are presented as mean ± SD of triplicates, and are representative of two independent experiments. *$P<0.05$ by Student's t-test.

(B) Effect of BAY61-3606 on cytokine productions by cDCs in response to LPS or ox-zymosan. WT cDCs were pretreated for 1 h with control vehicle (–) or indicated amounts of BAY 61-3606 (BAY), and then stimulated with LPS (100ng/mL) or Ox-zymosan (10 μg/mL). IL-6 and TNF-α levels in the cell culture supernatants were measured by ELISA. Data are presented as mean ± SD of triplicates, and are representative of two independent experiments. *$P<0.05$ by Student's t-test.
